# Illness Anxiety Disorder: A Case Report and Brief Review of the Literature

**DOI:** 10.7759/cureus.12897

**Published:** 2021-01-25

**Authors:** Eduardo D Espiridion, Anna Fuchs, Adeolu O Oladunjoye

**Affiliations:** 1 Psychiatry, Drexel University College of Medicine, Philadelphia, USA; 2 Psychiatry, West Virginia School of Osteopathic Medicine, Lewisburg, USA; 3 Psychiatry, West Virginia University School of Medicine, Martinsburg, USA; 4 Psychiatry, Philadelphia College of Osteopathic Medicine, Philadelphia, USA; 5 Psychiatry, Reading Hospital Tower Health, West Reading, USA; 6 Medicine, Drexel University College of Medicine, Philadelphia, USA; 7 Critical Care Medicine, Boston Children's Hospital, Boston, USA

**Keywords:** illness anxiety disorder, hypochondriasis, key words: anxiety

## Abstract

Illness anxiety disorder (IAD) is defined in the Diagnostic and Statistical Manual of Mental Disorders, 5th Edition (DSM-V) as the preoccupation with having or acquiring a serious illness, in the absence of somatic symptoms (or, if present, symptoms that are only mild in severity). Patients with IAD experience persistent anxiety or fear of having or acquiring a serious illness, which adversely affects their daily life. They remain unsatisfied with their physician’s reassurances to the contrary, mainly because their distress is created by the anxiety of the meaning, significance, and cause of the complaints and not necessarily due to the physical presentations. IAD remains a huge burden on both the health facility and for the managing healthcare provider. In this report, we present the case of a patient with IAD, which has been managed for the past five years with recurrent visits to the physician with no resolution of signs and symptoms. Despite extensive medical workup over this period, which repeatedly showed normal test results, the patient continued to have anxiety over his ill health and complained of recurrent mild somatic symptoms. After his most recent appointment, he got very upset and booked a flight to his home country to have a second opinion to validate his illness. Physicians are encouraged to build a therapeutic alliance with patients with IAD, rather than ordering expensive or unnecessary diagnostic tests or treatment.

## Introduction

Illness anxiety disorder (IAD), previously known as hypochondriasis, is defined in the Diagnostic and Statistical Manual of Mental Disorders, 5th Edition (DSM-V) as the preoccupation with having or acquiring a serious illness, in the absence of somatic symptoms (or, if present, symptoms that are only mild in severity) [1-2]. Patients with IAD experience a high level of anxiety about their health and they are easily alarmed by their health status [1]. They experience persistent anxiety or fear of having or acquiring a serious illness, adversely affecting their daily life [2-3]. They remain unsatisfied with their physician’s reassurances, mainly because their distress arises from the anxiety of the meaning, significance, and cause of the complaints and not necessarily from the physical presentation. These individuals engage in excessive health-related behaviors (e.g., repeated checks on the body for signs of illness) or exhibit maladaptive avoidance (e.g., avoiding doctor appointments and hospitals) [1]. The diagnosis requires the existence of the preoccupation for at least six months, but specific illnesses may change over that period of time [1]. The illness is not better explained by other mental disorders. There are two types of IAD: care-seeking type and care-avoidant type [1-2]. The care-seeking type involves those who frequently utilize the healthcare system and medical resources, including physician visits and undergoing multiple tests and procedures [2,4]. The care-avoidant type, on the other hand, refers to patients with severe anxiety who hold the belief that their physician visit or laboratory test will reveal life-threatening illnesses such as cancer [2,4]. In this report, we present a patient with IAD whose disease course has been characterized by recurrent visits to the physician in the past five years with no resolution of signs and symptoms.

## Case presentation

History of the presenting complaint

The patient was a 34-year-old male immigrant to the US from Asia who was admitted to the medical floor of a community hospital for the management of his medical problems. He had experienced a recurrent history of anxiety and mild somatic symptoms for the past five years. He reported sleeping difficulties, panic attacks, ruminative worries, muscle tension, body weakness, and chest discomfort. The patient had a prior psychiatric history and hospitalization for anxiety and depression. He had been admitted for anxiety and depression in a free-standing health facility five years ago and had been treated with medications. He had been prescribed sertraline and quetiapine but had self-discontinued because of tolerability issues. He had continued to be anxious with mild somatic symptoms. The patient denied any manic or psychotic symptoms or any neurological symptoms. The patient also denied any suicidal or homicidal ideations. He had no history of substance use or alcohol intake. He had a family history of anxiety disorder, described as an obsessive-compulsive disorder in his mother. In 2019, he had consulted a physician who had performed an extensive medical workup. All test results were unremarkable; he was reassured and his symptoms improved. During the two to three weeks prior to the current presentation, his anxiety and somatic symptoms had recurred and had subsequently worsened. He was then admitted for further investigation.

Examination

The mental status examination revealed a young man who was neat and well-groomed. He was cooperative, calm, and made appropriate eye contact. His speech was normal. He described his mood as anxious and his affect was constricted. His thought process was normal and linear. There was no suicidal or homicidal ideation. No delusions or hallucinations were reported. He was awake, alert, and oriented to time, place, people, and events. His judgment and insight were fair.

Investigation

He underwent a 12-lead electrocardiogram, which showed a ventricular rate of 80 beats/minute (reference range: 60-100 beats/minute), atrial rate of 80 beats/minute (reference range: 60-100 beats/minute), PR interval of 148 milliseconds (reference range: 120-200 milliseconds), QRS duration of 94 milliseconds (reference range: 80-100 milliseconds), QT interval of 350 milliseconds (reference range: 360-430 milliseconds), and QTC calculation (Bazett) of 403 milliseconds (reference range: ≤440 milliseconds) with normal sinus rhythm.

Other medical workups done revealed thyroid-stimulating hormone of 2.398 uIU/mL (reference range: 0.35-5.5 uIU/mL), hemoglobin A1c of 5.6% (reference range: <5.7%), and urinalysis showing no ketones or proteins. Lipid profile showed cholesterol of 182 milligrams per deciliter (reference range: <200 milligrams per deciliter), high-density lipoprotein of 39 milligrams per deciliter (reference range: <40 milligrams per deciliter), low-density lipoproteins of 103.8 milligrams per deciliter (reference range: <100 milligrams per deciliter), and a slightly elevated triglyceride of 196 milligrams per deciliter (reference range: <150 milligrams per deciliter). His basic metabolic panel was as follows: sodium of 140 millimoles per liter (reference range: 136-145 millimoles per liter), potassium of 4.1 millimoles per liter (reference range: 3.6-5.2 millimoles per liter), chloride of 104 millimoles per liter (reference range: 98-106 millimoles per liter), glucose of 93 milligrams per deciliter (reference range: 70-100 milligrams per deciliter), calcium of 9.3 milligrams per deciliter (reference range: 8.5-10.5 milligrams per deciliter), phosphorus of 4.0 milligrams per deciliter (reference range: 3.4-4.5 milligrams per deciliter), blood urea nitrogen of 9 milligrams per deciliter (reference range: 7-20 milligrams per deciliter), and creatinine of 1.0 milligrams per deciliter (reference range: 0.84-1.21 milligrams per deciliter). His complete blood count revealed a hematocrit of 48.9% (reference range: 38.3-48.6%), mean corpuscular volume of 84.7 femtoliters (reference range: 80-96 femtoliters), and red cell distribution width of 12.5% (reference range: 11.8-14.5%). All of these results were unremarkable. Also, an MRI of the brain without contrast was unremarkable. His vitamin B12 was unremarkable as well: 337 picograms per milliliter (reference range: 160-950 picograms per milliliter).

Both the hospitalist and cardiologist cleared him for any medical issues. Despite this reassurance, he got very upset and booked a flight to his home country to have a second opinion to validate his illness.

## Discussion

The cause of IAD is largely unknown. However, several risk factors have been implicated in the development of IAD. These include an underlying anxiety disorder, excess amount of time spent reviewing health-related materials (e.g., on the internet), history of previous serious childhood illness or illness of the patient's caregiver, family history of anxiety, and discussions and experience that involve labeling normal body sensations as pathological [2,3-5]. Our patient did not present with any of these risk factors; however, he had a history of anxiety and depression. These patients usually present with comorbidities such as anxiety and depression and need to be treated for these conditions [6]. What is not known is whether psychiatric history was a trigger to IAD, or if these psychiatric conditions coexisted in our patient from the onset. This patient had discontinued treatment for his anxiety and depression, which may have also triggered IAD in him.

IAD is a diagnosis of exclusion. It is important that a comprehensive examination and testing be conducted to exclude any organic disease before establishing a diagnosis of IAD [2,4]. Patients with IAD typically utilize several medical facilities. They are seen by multiple physicians with repeated negative tests each time they visit a health facility [1]. Frequently, the physician will carry out unnecessary and costly tests without achieving satisfying results; though the patient is deemed healthy, the anxiety persists [7]. Therefore, patients continue to visit multiple physicians due to their frustration with repeatedly normal or negative test results and unsuccessful physician reassurance. In the case of our patient, after several visits with physicians in the US, he got upset with his management and decided to travel to see another physician in his home country for a second opinion.

Patients with IAD are more often found in primary care centers than in mental health clinics, resulting in a delay in treatment and subsequent worsening of their psychiatric condition [7,8]. The primary care provider (PCP) is typically trained to identify physical symptoms that are not found in patients with IAD. This results in excessive use of scarce medical resources, worsening the depletion of the health resources apart from wasting the physician’s time and efforts [9]. It is not known how much money has been spent on this patient in total, but it is expected to run into several thousands of US dollars. It was estimated that 10-20% of the US medical budget is spent on patients with some form of somatization or IADs [10].

The most important aspect in the management of IAD is for the physician to establish a longitudinal trust relationship with the patient [11]. Communication should focus on empathy, open dialog, coordination of testing, and consistent delivery of messages [3]. The physician should first acknowledge their fears and concerns with the patient. Patients need to be reassured that although there is no specific treatment for the unexplained symptoms, the symptom is not fatal or catastrophic and that the physician will continue to work with patients on their path back to health and well-being. Holder-Perkins et al. have proposed that the physician should focus on psychosocial problems instead of the somatic concerns of the patient [8]. Patients can have regular follow-up visits with their PCP and psychiatrist to address new complaints, triggers, or stressors [4]. This will ultimately reduce unnecessary visits to the emergency department or other physicians.

Psychotherapy is the first-line treatment for IAD and has been shown to reduce symptoms associated with the condition [2]. This includes cognitive-behavioral therapy, which focuses on eradicating dysfunctional maladaptive cognitive beliefs by means of behavioral modification strategies [2,4]. Other therapy options include mindfulness-based cognitive therapy, group-therapies, attention training, and acceptance and commitment therapy [2,7]. Psychotropic medications have also been helpful in treating marked comorbidity of anxiety and depressive symptoms in patients with IAD [8]. Antidepressants such as selective serotonin reuptake inhibitors (SSRIs) and serotonin-norepinephrine reuptake inhibitors (SNRIs) have been proven to be effective in patients with IAD [2]. However, the challenges of prescribing these drugs include the patient’s misinterpretation of this as an attempt to dismiss their unexplained symptoms, as well as the side effects of these medications. Patients should be reassured about using medications and given very detailed information about the management plan during scheduled visits.

## Conclusions

Physicians are encouraged to build a therapeutic alliance with patients with IAD as they work with them on their path back to health. This is essential to reduce the tremendous burden on the healthcare system caused by the wastage of physician resources and ordering of expensive or unnecessary investigations while caring for patients with IAD.

## References

[1] (2013). Diagnostic and Statistical Manual of Mental Disorders: DSM-5.

[2] French JH, Hameed S (2020). Illness Anxiety Disorder. StatPearls.

[3] Scarella TM, Boland RJ, Barsky AJ (2019). Illness anxiety disorder: psychopathology, epidemiology, clinical characteristics, and treatment. Psychosom Med.

[4] Newby JM, Hobbs MJ, Mahoney AEJ, Wong SK, Andrews G (2017). DSM-5 illness anxiety disorder and somatic symptom disorder: Comorbidity, correlates, and overlap with DSM-IV hypochondriasis. J Psychosom Res.

[5] Alberts NM, Hadjistavropoulos HD, Sherry SB, Stewart SH (2016). Linking illness in parents to health anxiety in offspring: do beliefs about health play a role?. Behav Cogn Psychother.

[6] Noyes R Jr (1999). The relationship of hypochondriasis to anxiety disorders. Gen Hosp Psychiatry.

[7] Fallon BA, Ahern DK, Pavlicova M, Slavov I, Skritskya N, Barsky AJ (2017). A randomized controlled trial of medication and cognitive-behavioral therapy for hypochondriasis. Am J Psychiatry.

[8] Holder-Perkins V, Wise TN, Williams DE (2000). Hypochondriacal concerns: management through understanding. Prim Care Companion J Clin Psychiatry.

[9] Almalki M, Al-Tawayjri I, Al-Anazi A, Mahmoud S, Al-Mohrej A (2016). A recommendation for the management of illness anxiety disorder patients abusing the health care system. Case Rep Psychiatry.

[10] Ford CV (1986). The somatizing disorders. Psychosomatics.

[11] Starcevic V (2015). Hypochondriasis: treatment options for a diagnostic quagmire. Australas Psychiatry.

